# Supplementary material for: Heat adaptation of phage T7 under an extended genetic code
Source: Virus Evol. 2021 Dec 1;7(2):veab100. doi: 10.1093/ve/veab100 (PMC8923235; doi:10.1093/ve/veab100)
Supplement: veab100_Supp [file veab100_supp.zip › Sequencing_Analysis.zip!/Sequencing_Analysis/A3/evidence/JC_55.html]

BRESEQ :: Evidence


| Predicted mutation | | | | | | |
| --- | --- | --- | --- | --- | --- | --- |
| evidence | seq id | position | mutation | annotation | gene | description |
| MC JC | NC\_001604.1 | 1,253 | Δ1,489 bp | intergenic (–/–) | – / – | –/– |

| Missing coverage evidence... | | | | | | | | | | |
| --- | --- | --- | --- | --- | --- | --- | --- | --- | --- | --- |
|  |  |  | seq id | start | end | size | ←reads | reads→ | gene | description |
| \* | \* | ÷ | NC\_001604.1 | 1253 | 2741 | 1489 | 1819 [50] | [67] 1835 | *–/–* | –/– |

| New junction evidence | | | | | | | | | | | |
| --- | --- | --- | --- | --- | --- | --- | --- | --- | --- | --- | --- |
|  | | seq id | position | reads (cov) | reads (cov) | score | skew | freq | annotation | gene | product |
| \* | ? | NC\_001604.1 | = 1252 | 50 (0.030) | 1744 (1.030) | 316/580 | 0.1 | 96.8% | intergenic (–/–) | *–/–* | –/– |
|  | ? | NC\_001604.1 | 2742 = | 66 (0.040) |  |  |  |  | intergenic (–/–) | *–/–* | –/– |

Only 100 of 1832 total aligned reads displayed.  

`ACTTACAACAACGTTTTCGACCACGCTTACGAAATGCTGAAAGAAAACATCCGTTATGATGACATCCGTGACACTGATGACCTGCACGATGCTATTCACATGGCTGCCGATAATGCAGTTCCGCACTACTACGCTGACATCTTTAGCGTAATGGCAAGTGAGGGCATTGACCTTGAGTTCGAAGACTCTGGTCTGATGCCTGACACCAAGGACGTAATCCGCATCCTGCAAGCGCGTATCTATGAGCAATTAACGATTGACCTCTGGGAAGACGCAGAAGACTTGCTCAATGAATACTTG‑‑‑‑‑‑‑‑‑G‑‑‑‑‑‑‑‑‑A‑‑‑‑‑‑‑‑‑GGAAGTCG‑‑‑‑‑‑‑‑‑‑‑‑‑‑‑‑‑‑‑‑‑‑‑‑‑‑‑‑‑‑‑‑‑‑‑‑‑‑‑‑‑‑‑‑‑‑‑‑‑‑‑‑‑‑‑‑‑‑‑‑‑‑‑‑‑‑‑‑‑‑‑‑‑‑‑‑‑‑‑‑‑‑‑‑‑‑‑‑‑‑‑‑‑‑‑‑‑‑‑‑‑‑‑‑‑‑‑‑‑‑‑‑‑‑‑‑‑‑‑‑‑‑‑‑‑‑‑‑‑‑‑‑‑‑‑‑‑‑‑‑‑‑‑‑‑‑‑‑‑‑‑‑‑‑‑‑‑‑‑‑‑‑‑‑‑‑‑‑‑‑‑‑‑‑‑‑‑‑‑‑‑‑‑‑‑‑‑‑‑‑‑‑‑‑‑‑‑‑‑‑‑‑‑‑‑‑‑‑‑‑‑‑‑‑‑‑‑‑‑‑‑‑‑‑‑‑‑‑‑‑‑‑‑‑‑‑‑‑‑‑‑‑‑‑‑‑‑‑‑‑‑‑‑‑‑‑‑‑‑‑‑‑‑‑‑‑‑‑‑‑‑‑‑‑‑‑‑‑‑‑‑‑‑‑‑‑‑‑‑‑‑‑‑‑‑‑‑‑‑‑‑‑‑‑‑‑‑‑‑  >  NC_001604.1/943‑1252`  
`‑‑‑‑‑‑‑‑‑‑‑‑‑‑‑‑‑‑‑‑‑‑‑‑‑‑‑‑‑‑‑‑‑‑‑‑‑‑‑‑‑‑‑‑‑‑‑‑‑‑‑‑‑‑‑‑‑‑‑‑‑‑‑‑‑‑‑‑‑‑‑‑‑‑‑‑‑‑‑‑‑‑‑‑‑‑‑‑‑‑‑‑‑‑‑‑‑‑‑‑‑‑‑‑‑‑‑‑‑‑‑‑‑‑‑‑‑‑‑‑‑‑‑‑‑‑‑‑‑‑‑‑‑‑‑‑‑‑‑‑‑‑‑‑‑‑‑‑‑‑‑‑‑‑‑‑‑‑‑‑‑‑‑‑‑‑‑‑‑‑‑‑‑‑‑‑‑‑‑‑‑‑‑‑‑‑‑‑‑‑‑‑‑‑‑‑‑‑‑‑‑‑‑‑‑‑‑‑‑‑‑‑‑‑‑‑‑‑‑‑‑‑‑‑‑‑‑‑‑‑‑‑‑‑‑‑‑‑‑‑‑‑‑‑‑‑‑‑‑‑‑‑‑‑‑‑‑‑‑‑‑‑‑‑‑‑‑‑‑‑‑‑‑‑‑‑‑‑‑‑‑‑‑‑‑‑‑‑‑‑‑‑‑‑‑‑‑‑‑‑‑‑‑‑‑‑‑‑‑g‑‑‑‑‑‑‑‑‑a‑‑‑‑‑‑‑‑‑ggaagtcg‑‑‑‑‑‑‑C‑‑ACGACAGAAAGAAATTGACCGCGCTAAGGCCCGTAAAGAACGTCACGAGGGGCGCTTAGAGGCACGCAGATTCAAA‑CGTCGCAACCGCAAGGCACGTAAAGCACACAAAGCTAAGCGCGAAAGAATGCTTGCTGCGTGGCGATGGGCTGAACGTCAAGAACGGCGTAACCATGAGGTAGCTGTAGATGTACTAGGAAGAACCAATAACGCTATGCTCTGGGTCAACATGTTCTCTGGGGACTTTAAGGCGCTTGAGGAACGAATCGCGCTGCACTGGCGTAATGCTGACCGGATGGCT  >  NC_001604.1/2742‑3040`  

                                                                                                                                                                                                                                                                                                                                                                                                                                                                                                                                                                                                                                                                        
   `TACACCAACGTTTTCGACCACGCTTACGAAATGCTGAAAGAAAACATCCGTTATGATGACATCCGTGACACTGATGACCTGCACGATGCTATTCACATTGCTGCCGATAATTCAGTTCCGCACTACTACGCTGACATCTTTAGCGTAATGGCAAGTGAGGGCACTGATCTTGAGTTCGAAGACTCTGGTCTGATGCCTGACACCAAGGACGTAATCCGCATCCTGCAAGCGCGTAACTATGAGCAATTAACGATTGACCTCTGGGAAGACGCAGAAGACTTGCTCAATGAATACTTG‑‑‑‑‑‑‑‑‑G‑‑‑‑‑‑‑‑‑A‑‑‑‑‑‑‑‑‑GG                                                                                                                                                                                                                                                                                                                             <  1:137341/301‑1`  
       `ACAACGTTTTCGACCACGCTTACGAAATGCTGAAAGAAAACATCCGTTATGATGACATCCGTGACACTGATGACCTGCACGATGCTATTCACATGGCTGCCGATAATGCAGTTCCGCACTACTACGCTGACATCTTTAGCGTAATGGCAAGTTATGGCATTGACCTTGAGTTCGAAGACTCTGGTCTGATGCCTGACACCAAGGACGTAATCCGCATCCTGCAAGCGCGTATCTATGAGCAATTAACGATTGACCTCTGGGAAGACGCAGAAGACTTGCTCAATGAATACTTG‑‑‑‑‑‑‑‑‑G‑‑‑‑‑‑‑‑‑A‑‑‑‑‑‑‑‑‑GGAAGT                                                                                                                                                                                                                                                                                                                         <  1:131372/301‑1`  
        `CAACGTTTTCGACCACGCTTACGAAATGCTGAAAGAAAACATCCGTTATGATGACATCCGTGACACTGATGACCTGCACGATGCTATTCACATGGCTGCCGATAATGCAGTTCCGCACTACTACGCTGACATCTTTAGCGTAATGGCAAGTGAGGGCATTGACCTTGAGTTCGAAGACTCTGGTCTGATGCCTGACACCAAGGACGTAATCCGCATCCTGCAAGCGCGTATCTATGAGCAATTAACGATTGACCTCTGGGAAGACGCAGAAGACTTGCTAAATGAATACTTG‑‑‑‑‑‑‑‑‑G‑‑‑‑‑‑‑‑‑A‑‑‑‑‑‑‑‑‑GGAAGTC                                                                                                                                                                                                                                                                                                                        <  1:38633/301‑1`  
            `GTTTTCGACCACGCTTACGAAATGCTGAAAGAAAACATCCGTTATGATGACATCCGTGACACTGATGACCTGCACGATGCTATTCACATGGCTGCCGATAATGCAGTTCCGCACTACTACGCTGACATCTTTAGCGTAATGGCAAGTGAGGGCATTGACCTTGAGTTCGAAGACTCTGGTCTGATGCCTGACACCAAGGACGTAATCCGCATCCTGCAAGCGCGTATCTATGAGCAATTAACGATTGACCTCTGGGAAGACGCAGAAGACTTGCTCAATGAATACTTG‑‑‑‑‑‑‑‑‑G‑‑‑‑‑‑‑‑‑A‑‑‑‑‑‑‑‑‑GGAAGTCG‑‑‑‑‑‑‑C‑‑AC                                                                                                                                                                                                                                                                                                           <  1:276594/301‑1`  
                 `CGACCACGCTTACGAAATGCTGAAAGAAAACATCCGTTATGATGACATCCGTGACACTGTTGACCTGCACGATGCTATTCACATGGCTGCCGATAATGCAGTTCCGCACTACTACGCTGACATCTTTAGCGTAATGGCAAGTGAGGGCATTGACCTTGAGTTCGAAGACTCTGGTCTGATGCCTGACACCAAGGACGTAATCCGCATCCTGCAAGCGCGTATCTATGAGCAATTAACGATTGACCTCTGGGAAGACGCAGAAGACTTGCTCAATGAATACTTG‑‑‑‑‑‑‑‑‑G‑‑‑‑‑‑‑‑‑A‑‑‑‑‑‑‑‑‑Gctgtctcctatacac                                                                                                                                                                                                                                                                                                               >  1:10977/1‑286`  
                       `CGCTTACGAAATGCTGAAAGAAAACATCCGTTATGATGACATCCGTGACACTGATGACCTGCACGATGCTATTCACATGGCTGCCGATAATGCAGTTCCGCACTACTACGCTGACATCTTTAGCGTAATGGCAAGTGAGGGCATTGACCTTGAGTTCGAAGACTCTGGTCTGATGCCTGACACCAAGGACGTAATCCGCATCCTGCAAGCGCGTATCTATGAGCAATTAACGATTGACCTCTGGGAAGACGCAGAAGACTTGCTCAATGAATACTTG‑‑‑‑‑‑‑‑‑G‑‑‑‑‑‑‑‑‑A‑‑‑‑‑‑‑‑‑GGAAGTCG‑‑‑‑‑‑‑C‑‑ACGACctgtctct                                                                                                                                                                                                                                                                                                >  1:327210/1‑293`  
                         `CTTACGAAATGCTGAAAGAAAACATCCGTTATGATGACATCCGTGACACTGATGACCTGCACGATGCTATTCACATGGCTGCCGATAATGCAGTTCCGCACTACTACGCTGACATCTTTAGCGTAATGGCAAGTGAGGGCATTGACCTTGAGTTCGAAGACTCCGGTCTGATGCCTGACACCAAGGACGTAATCCGCATCCTGCAAGCGCGTATCTATGAGCAATTAACGATTGACCTCTGGGAAGACGCAGAAGACTTGCTCAATGAATACTTG‑‑‑‑‑‑‑‑‑G‑‑‑‑‑‑‑‑‑A‑‑‑‑‑‑‑‑‑GGAAGTCG‑‑‑‑‑‑‑C‑‑ACGACAGAAAGAAAT                                                                                                                                                                                                                                                                                              <  1:144465/301‑1`  
                             `ctAAATGATGAAAGCAAACATCCGTTATGATTACATCCGTAACAGTGATCACCTGCACGATGCTATTCACGTTGCTTCCGATAATGCATTTCCGCACTCCTACGCTTACATCTTTAGCATAATGGCAAGTGAGGGCATTGACCTTGAGTTCGAAGACTCTGGTCTGATGCCTGACACCAAGGACGTAATCCGCATCCTGCAAGCGCGTATCTATGAGCAATTAACGATTGACCTCTGGGAAGACGCAGAAGACTTGCTCAATGAATACTTG‑‑‑‑‑‑‑‑‑G‑‑‑‑‑‑‑‑‑A‑‑‑‑‑‑‑‑‑GGAAGTCG‑‑‑‑‑‑‑C‑‑ACGACAGAAAGAAATTGAC                                                                                                                                                                                                                                                                                          <  1:81564/299‑1`  
                                              `ACATCCGTTATGATGACAGCCGTGACACTGATGACCTGCACGATGCTATTCACATGGCTGCCGATAATGCAGTTCCGCACTACTACGCTGACATATTTAGCGTAATGGCAAGTGAGGGCATTGACCTTGAGTTCGAAGACTCTGGTCTGATGCCTGACACCAAGGACGTAATCCGCATCCTGCAAGCGCGTATCTATGAGCAATTAACGATTGACCTCTGGGAAGACGCAGAAGACTTGCTCAATGAATACTTG‑‑‑‑‑‑‑‑‑G‑‑‑‑‑‑‑‑‑A‑‑‑‑‑‑‑‑‑GGAAGTCG‑‑‑‑‑‑‑C‑‑ACGACAGAAAGAAATTGACCGCGCTAAGGCCCGTAA                                                                                                                                                                                                                                                                         <  1:32963/301‑1`  
                                                 `tcaGTTATGATGACATCCGTGACACTGATGACCTGCACGATGCTATTCACATGGCTGCCGATAATGCAGGTCCGCACTACTACGCTGACATCTTTAGCGTAAGGGCAAGTGAGGGCATTGACCTTGAGTTCGAAGACTCTGGGCTGATGCCTGACACCAAGGACGTAATCCGCATCCTGCAAGCGCGTATCTATGAGCAATTAACGATCGACCTCTGGGAAGACGCAGAAGACTTGCTCAATGAATACTTGGAGGAAGTCG‑‑‑‑‑‑‑‑‑A‑‑‑‑‑‑‑‑‑GGAAGTCG‑‑‑‑‑‑‑C‑‑ACGACAGAAAGAAATTGACCGCGCTAAGGC                                                                                                                                                                                                                                                                               <  1:53625/298‑1`  
                                                 `TCCGTTATGATGACATCCGTGACACTGATGACCTGCACGATGCTATTCACATGGCTGCCGATAATGCAGTTCCGCACTACTACGCTGACATCTTTAGCGTAATGGCAAGTGAGGGCATTGACCTTGAGTTCGAAGACTCTGGTCTGATGCCTGACACCAAGGACGTAATCCGCATCCTGCAAGCGCGTATCTATGAGCAATTAACGATTGACCTCTGGGAAGACGCAGAAGACTTGCTCAATGAATACTTG‑‑‑‑‑‑‑‑‑G‑‑‑‑‑‑‑‑‑AGGAAGTCGGGGAAGTCG‑‑‑‑‑‑‑C‑‑ACGACAGAAAGAAATTGACCGCGCTAAGGC                                                                                                                                                                                                                                                                               <  1:282845/301‑1`  
                                                         `acaGACATCCGTGACACTGATGACCTGCACGATGCTATTCACATGGCTGCCGATAATGCAGTTCCGCACTACTACGCTGACATCTTTAGCGTAATGGCAAGTGAGGGCATTGACCTTGAGTTCGAAGACTCTGGTCTGATGCCTGACACCAAGGACGTAATCCGCATCCTGCAAGCGCGTATCTATGAGCAATTAACGATTGACCTCTGGGAAGAAGCAGAAGACTTGCTCAATGAATACTTGGAGGAAGTCG‑‑‑‑‑‑‑‑‑A‑‑‑‑‑‑‑‑‑GGAAGTCG‑‑‑‑‑‑‑C‑‑ACGACAGAAAGAAATTGACCGCGCTAAGGCCCGTAAAG                                                                                                                                                                                                                                                                       <  1:218813/298‑1`  
                                                         `GATGACATCCGTGACACTGATGACCTGCACGATGCTATTCACATGGCTGCCGATAATGCAGTTCCGCACTACTACGCTGACATCTTTAGCGTAATGGCAAGTGAGGGCATTGACCTTGAGTTCGAAGACTCTGGTCTGATGCCTGACACCAAGGACGTAATCCGCATCCTGCAAGCGCGTATCTATGAGCAATTAACGATTGACCTCTGGGAAGACGCAGAAGACTTGCTCAATGAATACTTGGAGGAAGTCG‑‑‑‑‑‑‑‑‑A‑‑‑‑‑‑‑‑‑GGAAGTCG‑‑‑‑‑‑‑C‑‑ACGACAGAAAGAAATTGACCGCGCTAAGGCtgtctctt                                                                                                                                                                                                                                                                       >  1:221772/1‑293`  
                                                         `GATGACATCCGTGACACTGATGACCTGCACGATGCTATTCACATGGCTGCCGATAATGCAGTTCCGCACTACTACGCTGACATCTTTAGCGTAATGGCAAGTGAGGGCATTGACCTTGAGTTCGAAGACTCTGGTCTGATGCCTGACACCAAGGACGTAATCCGCATCCTGCAAGCGCGTATCTATGAGCAATTAACGATTGACCTCTGGGAAGACGCAGAAGACTTGCTCAATGAATACTTG‑‑‑‑‑‑‑‑‑G‑‑‑‑‑‑‑‑‑A‑‑‑‑‑‑‑‑‑GGAAGTCG‑‑‑‑‑‑‑C‑‑ACGACAGAAAGAAATTGACCGCGCTAAGGCCCGTAAAGAACGTCACG                                                                                                                                                                                                                                                              <  1:16338/301‑1`  
                                                                `TCCGTGACACTGATGACCTGCACGATGCTATTCACATGGCTGCCGATAATGCAGTTCCGCACTACTACGCTGACATCTTTAGCGTAATGGCAAGTGAGGGCATTGACCTTGAGTTCGAAGACTCTGGTCTGATGCCTGACACCAAGGACGTAATCCGCATCCTGCAAGCGCGTATCTATGAGCAATTAACGATTGACCTCTGGGAAGACGCAGAAGACTTGCTCAATGAATACTTGGAAGAAGTCG‑‑‑‑‑‑‑‑‑A‑‑‑‑‑‑‑‑‑GGAAGTCG‑‑‑‑‑‑‑C‑‑ACGACAGAAAGACATTGACCGCGCTAAGGCCCGTAAAGACCGTCA                                                                                                                                                                                                                                                                >  1:267412/1‑301`  
                                                                      `accCTGATTACCTGCACTATGCTATTCACATGGCTGCCGATAATGCAGTTCCGCACTACTACGCTGACATCTTTAGCGTAATGGCAAGTGAGGGCATTGACCTTGAGTTCGAAGACTCTGGTCTGATGCCTGACACCAAGGACGTAATCCGCATCCTACAAGCGCGTATCTATGAGCAATTAACGATTGACCTCTGGGAAGACGCAGAAGACTTGCTCAATGAATACTTG‑‑‑‑‑‑‑‑‑G‑‑‑‑‑‑‑‑‑A‑‑‑‑‑‑‑‑‑GGAAGTCG‑‑‑‑‑‑‑C‑‑ACGACAGAAAGAAATTGACCGCGCTAAGGCCCGTAAAGAACGTCACGAGGGGCGCTTAGA                                                                                                                                                                                                                                                 <  1:7195/298‑1`  
                                                                         `CTGATGACCTGCACGATGCTATTCACATGGCTGCCGATAATGCAGTTCCGCACTACTACGCTGACATCTTTAGCGTAATGGCAAGTGAGGGCATTGACCTTGAGTTCGAAGACTCTGGTCTGATGCCTGACACCAAGGACGTAATCCGCATCCTGCAAGCGCGTATCTATGAGCAATTAACGATTGACCTCTGGGAAGACGCAGAAGACTTGCTCAATGAATACTTG‑‑‑‑‑‑‑‑‑G‑‑‑‑‑‑‑‑‑A‑‑‑‑‑‑‑‑‑TGAAGTCG‑‑‑‑‑‑‑C‑‑ACGACAGAAAGAAATTGACCGCGCTAAGGCCCGTAAAGAACGTCCCGAGGGGCGCTTAGAGGC                                                                                                                                                                                                                                              >  1:26469/1‑301`  
                                                                              `aACCGGCACGATGCCAGTCACATGGCGGCCGATAATGCAGTACCGCACTACTACGCTGACATCTTTAGCGTAATGGCAAGTGAGGGCATTGACCTTGAGTTCGAATACTCTGGTCTGATGCCTGACACCAAGGACGTAATCCGCATCCTGCAAGCGCGTATCTATGAGCAATTAACGGTTGACCTCTGGGAAGACGCAGAAGACTTGCTCAATGAATACTTGGAGGAAGTCG‑‑‑‑‑‑‑‑‑A‑‑‑‑‑‑‑‑‑GGAAGTCG‑‑‑‑‑‑‑C‑‑ACGACAGAAAGAAATTGACCGCGCTAAGGCCCGTAAAGAACGTCACGAGGGGCGCTTAG                                                                                                                                                                                                                                                  <  1:238710/300‑1`  
                                                                              `GACCTGCACGATGCTATTCACATGGCTGCCGATAATGCAGTTCCGCACTACTACGCTGACATCTTTAGCGTAATGGCAAGTGAGGGCATTGACATTGAGTTCGAAGACTCTGGTCTGATGCCTGACACCAAGGACGTAATCCGCATCCTGCAAGCGCGTATCTATGAGCAATTAACGATGGACCTCTGGGAAGACGCAGAAGACTTGCTCAATGAATACTTG‑‑‑‑‑‑‑‑‑G‑‑‑‑‑‑‑‑‑A‑‑‑‑‑‑‑‑‑GGAAGTCG‑‑‑‑‑‑‑C‑‑ACGACAGAAAGAAATTGACCGCGCTAAGGCCCGTAAAGAACGGCACTAGGGGGGCTTGGAGGCAAGCA                                                                                                                                                                                                                                         >  1:331823/1‑301`  
                                                                                      `aGATGCTATTCAAATGGCGGCCGATAATGCAGTTCCGCACTACTACGCTGACATCTTTAGCGTAATGGCAAGTGAGGGCATTGACCTTGAGTTCGAAGACTCTGGTCTGATGCCTGACACCAAGGACGTAATCCGCATCCTGCAAGCGCGTATCTATGAGCAATTAACGATTGACCTCTGGGAAGACGCAGAAGACTTGCTCAATGAATACTTG‑‑‑‑‑‑‑‑‑G‑‑‑‑‑‑‑‑‑A‑‑‑‑‑‑‑‑‑GGAAGTCG‑‑‑‑‑‑‑C‑‑ACGACAGAAAGAAATTGACCGCGCTAAGGCCCGTAAAGAACGTCACGAGGGGCGCTTAGAGGCACGCAGATTCAAA                                                                                                                                                                                                                                 <  1:44305/300‑1`  
                                                                                       `aagagacagCACATGGCTGCCGATAATGCAGTTCCGCACTACTACGCTGACATCTTTAGCGTAATGGCAAGTGAGGGCATTGACCTTGAGTTCGAAGACTCTGGGCTGATGCCTGACACCAAGGACGTAATCCGCATCATGCAAGCGCGTATCTATGAGCAATGAACGATTGACCTCTGGGAAGACGCAGAAGACTTGCTCAATGAATACTTG‑‑‑‑‑‑‑‑‑G‑‑‑‑‑‑‑‑‑A‑‑‑‑‑‑‑‑‑GGAAGTCG‑‑‑‑‑‑‑C‑‑ACGACAGAAAGAAATTGACCGCGCTAAGGCCCGTAAAGAACGTCACGAGGGGCACTTAGAGGCACGCAGATTCAAA‑C                                                                                                                                                                                                                               <  1:141848/292‑1`  
                                                                                       `GATGCTATTCACATGGCTGCCGATAATGCAGTTCCGCACTACTACGCTGACATCTTTAGCGTAATGGAAAGTGAGGGCATTGACCTTGAGTTCGAAGACTCTGGTCTGATGCCTGACACCAAGGACGTAATCCGCATCCTGCAAGCGCGTATCTATGAGCAATTAACGATTGACCTCTGGGAAGACGCAGAAGACTTGCTCAATGAATACTTG‑‑‑‑‑‑‑‑‑G‑‑‑‑‑‑‑‑‑A‑‑‑‑‑‑‑‑‑GGAAGTCG‑‑‑‑‑‑‑C‑‑ACGACAGAAAGAAATTGACCGCGCTAAGGCCCGTAAAGAACGTCACGAGGGGCGCTTAGAGGCACGCAGATTCAAA‑C                                                                                                                                                                                                                               <  1:305362/301‑1`  
                                                                                        `ATGCTATTCACATGGCTGCCGATAATGCAGTTCCGCACTACTACGCTGACATCTTTAGCGTAATGGCAAGTGAGGGCATTGACCTTGAGTTCGAAGACTCTGGTCTGATGCCTGACACCAAGGACGTAATCCGCATCCTGCAAGCGCGTATCTATGAGCAATTAACGATTGACCTCTGGGAAGACGCAGAAGACTTGCTCAATGAATACTTG‑‑‑‑‑‑‑‑‑G‑‑‑‑‑‑‑‑‑A‑‑‑‑‑‑‑‑‑GGAAGTCG‑‑‑‑‑‑‑C‑‑ACGACAGAAAGAAATTGACCGCGCTAAGGCCCGTAAATAACGTCACGAGGGGCGCTTAGAGGCACGCAGATTCAAA‑CG                                                                                                                                                                                                                              >  1:124976/1‑301`  
                                                                                             `ATTCACATGGCTGCCGATAATGCAGTTCCGCACTACTACGCTGACATCTTTAGCGTAATGGCAAGTGAGGGCATTGACCTTGAGTTCGAAGACTCTGGTCTGATGCCTGACACCAATGACGTAATCCGCATCCTGCAAGCGCGTATCTATGAGCAATTAACGATTGACATCTGGGAAGACGCAGAAGACTTGCTCAATGAATACTTG‑‑‑‑‑‑‑‑‑G‑‑‑‑‑‑‑‑‑A‑‑‑‑‑‑‑‑‑GGAAGTCG‑‑‑‑‑‑‑C‑‑AAGACAGAAAGAAATTGACCGCGCTAAGGCCCGTAAAGAAAGTCACGAGCGGCGCTTAGAGGCACGCAGATTCAAA‑CTTCCCA                                                                                                                                                                                                                         >  1:317481/1‑301`  
                                                                                               `aCACATGGCTGCCGATAAGGAAGTTCCGGACGACTACGCTGACATCATTAGCGTAATGGCAAGGGAGGGCATTGACCTTGAGTTCGAAGACTCTGGTCTGATGCCTGACACCAAGGACGTAATCCGCAGCCTGCAAGCGCGTATCTAGGAGCAATTAACGATTGACCTCTGGGAAGACGCAGAAGACTTGCTCAAGGAATAATTG‑‑‑‑‑‑‑‑‑G‑‑‑‑‑‑‑‑‑A‑‑‑‑‑‑‑‑‑GGAAGTCG‑‑‑‑‑‑‑C‑‑ACGACAGAAAGAAATTGACCGCGCTAAGGCCCGTAAAGAACGTCACGAGGGGCGCTTAGAGGCACGCAGATGCAAA‑CGTCGCAAC                                                                                                                                                                                                                       <  1:149784/300‑1`  
                                                                                                       `tgtataagagacagGTTCCGCACTACTACGCTGACATCTTTAGCGTAATGGCAAGTTAGGGCATTGACCTTGAGTTCGAAGACTCTGGTCTGATGCCTGACACCAAGGACGTAATCCGCATCCTGCAAGCGCGTATCTATGAGCAATTAACGATTGACCTCTGGGAAGACGCAGAAGACTTGCTCAATGAATACTTG‑‑‑‑‑‑‑‑‑G‑‑‑‑‑‑‑‑‑A‑‑‑‑‑‑‑‑‑GGAAGTCG‑‑‑‑‑‑‑C‑‑ACGACAGAAAGAAATTGACCGCGCTAAGGCCCGTAAAGAACGTCACGAGGGGCGCTTAGAGGCACGCAGATTCAAA‑CGTCGCAACCGCAAGGC                                                                                                                                                                                                               <  1:104142/287‑1`  
                                                                                                          `CCGATAATGCAGTTCCGCACTACTACGCTGACATCTTTAGCGTAATGGCAAGTGAGGGCATTGACCTTGAGTTCGAAGACTCTGGTCTGATGCCTGACACCAAGGACGTAATCCGCATCCTGCAAGCGCGTATCTATGAGCAATTAACGATTGACCTCTGGGAAGACGCAGAAGACTTGCTCAATGAATACTTG‑‑‑‑‑‑‑‑‑G‑‑‑‑‑‑‑‑‑A‑‑‑‑‑‑‑‑‑GGAAGTCG‑‑‑‑‑‑‑C‑‑ACGACAGAAAGAAATTGACCGCGCTAAGGCCCGTAAAGAACGTCACGAGGGGCGCTTAGAGGCACGCAGATTCAAA‑CGTCGCAACCGCAAGGCACG                                                                                                                                                                                                            >  1:86204/1‑301`  
                                                                                                                `ATGCAGTTCCGCACTACTACGCTTACATCTTTAGCGTAATGGCAAGTGAGGGCATTGACCTTGAGTTCGAAGACTCTGGTCTGATGCCTGACACCAAGGACGTAATCCGCATCCTGCAAGCGCGTATCTATGAGCAATTAACGATTGACCTCTGGGAAGACGCAGAAGACTTGCTCAATGAATACTTG‑‑‑‑‑‑‑‑‑G‑‑‑‑‑‑‑‑‑A‑‑‑‑‑‑‑‑‑TGAAGTCG‑‑‑‑‑‑‑C‑‑ACGACAGAAAGAAATTGACCGCGCTACGGCCCGTAAAGAACGTCACGAGGGGCGCTTAGAGGCACGCAGATTCAAA‑CGTCtgtctcttatacacatctccga                                                                                                                                                                                                      >  1:290294/1‑279`  
                                                                                                                `ATGCAGTTCCGCACTACTACGCTGACATCTTTAGCGTAATGGCAAGTGAGGGCATTGACCTTGAGTTCGAAGACTCTGGTCTGATGCCTGACACCAAGGACGTAATCCGCATCCTGCAAGCGCGTATCTATGAGCAATTAACGATTGACCTCTGGGAAGACGCAGAAGACTTGCTCAATGAATACTTGGAGGAAGTCG‑‑‑‑‑‑‑‑‑A‑‑‑‑‑‑‑‑‑GGAAGTCG‑‑‑‑‑‑‑C‑‑ACGACAGAAAGAAATTGACCGCGCTAAGGCCCGTAAAGAACGTCACGAGGGGCGCTTAGAGGCACGCAGATTCAAA‑CGTCGCAACCGCAAGGC                                                                                                                                                                                                               >  1:75324/1‑301`  
                                                                                                                `ATGCAGTTCCGCACTACTACGCTGACATCTTTAGCGTAATGGCAAGTGAGGGCATTGACCTTGAGTTCGAAGACTCTGGTCTGATGCCTGACACCAAGGACGTAATCCGCATCCTGCAAGCGCGTATCTATGAGCAATTAACGATTGACCTCTGGGAAGACGCAGAAGACTTGCTCAATGAATACTTG‑‑‑‑‑‑‑‑‑G‑‑‑‑‑‑‑‑‑A‑‑‑‑‑‑‑‑‑GGAAGTCG‑‑‑‑‑‑‑C‑‑CCGACAGAAAGAAATTGACCGCGCTAAGGCCCGTAAAGAACGTCACGAGGGGAGCTTAGAGGCACGCAGATTCAAA‑CGTCGCAACCGCAActgtctcttata                                                                                                                                                                                                      >  1:178110/1‑289`  
                                                                                                                             `CTACTACGCTGACATCTTTAGCGTAATGGCAAGTGAGGGCATTGACCTTGAGTTCGAAGACTCTGGTCTGATGCCTGACACCAAGGACGTAATCCGCATCCTGCAAGCGCGTATCTCTGAGCAATTAACGATTGACCTCTGGGAAGACGCAGAAGACTTGCTCAATGAATCCTTG‑‑‑‑‑‑‑‑‑G‑‑‑‑‑‑‑‑‑A‑‑‑‑‑‑‑‑‑GGAAGTCG‑‑‑‑‑‑‑C‑‑ACGACAGAAAGAAATTGACCGCGCTAAGGCCCGTAAAGCACGTCACGAGGGGCGCTTAGAGGCACGCAGATTCAAA‑CGTCGCAACCGCCAGGCCCGTAAAGCACACTAAGCTAAG                                                                                                                                                                                         >  1:228009/1‑301`  
                                                                                                                             `CTACTACGCTGACATCTTTAGCGTAATGGCAAGTGAGGGCATTGACCTTGAGTTCGAAGACTCTGGTCTGATGCCTGACACCAAGGACGTAATCCGCATCCTGCAAGCGCGTATCTATGAGCAATTAACGATTGACCTCTGGGAAGACGCAGAAGACTTGCTCAATGAATACTTGGAGGAAGTCG‑‑‑‑‑‑‑‑‑A‑‑‑‑‑‑‑‑‑GGAAGTCG‑‑‑‑‑‑‑C‑‑ACGACAGAAAGAAATTGACCGCGCTAAGGCCCGTAAAGAACGTCACGAGGGGCGCTTAGAGGCACGCAGATTCAAA‑CGTCGCAACCGCAAGGCACGTAAAGCACAC                                                                                                                                                                                                  >  1:300907/1‑301`  
                                                                                                                             `CTACTACGCTGACATCTTTAGCGTAATGGCAAGTGAGGGCATTGACCTTGAGTTCGAAGACTCTGGTCTGATGCCTGACACCAAGGACGTAATCCGCATCCTGCAAGCGCGTATCTATGAGCAATTAACGATTGACCTCTGGGAAGACGCAGAAGACTTGCTCAATGAATACTTG‑‑‑‑‑‑‑‑‑G‑‑‑‑‑‑‑‑‑A‑‑‑‑‑‑‑‑‑GGAAGTCG‑‑‑‑‑‑‑C‑‑ACGACAGAAAGAAATTGACCGCGCTAAGGCCCGTAAAGAACGTCACGAGGGGCGCTTAGAGGCACGCAGATTCAAA‑CGTCGCAACCGCAAGGCACGTAAAGCACACATAGCTAAG                                                                                                                                                                                         >  1:258954/1‑301`  
                                                                                                                                    `GCTGATATCTTTAGCGTAATGGCAAGTGAGGGCATTGACCTTGAGTTCGAAGACTCTGGTCTGATGCCTGACACACAGGACGTAATCCGCATCCTGCAAGCGCGTATCTATGAGCCATTAACGATTGACCTCTGGGAAGACGCAGAAGACTTGCTCAATGCATCCTTG‑‑‑‑‑‑‑‑‑G‑‑‑‑‑‑‑‑‑A‑‑‑‑‑‑‑‑‑GGAAGTCG‑‑‑‑‑‑‑C‑‑ACGACCGAACGAAATTGACCGCGCTAAGGCCCGTAAAGAACGTCACGAGGTGCGCTTAGAGGCACGCAGATTCACA‑CGTCGCAACCGCACGGCACGTAAAGCACATACAGCTAAGCGagcaa                                                                                                                                                                                  >  1:2425/1‑296`  
                                                                                                                                              `ggagatgggtataagagacaGGCATTGACCTTGAGTTCGAAGTCTCTGGTCTGATGCCTGACACCAAGGACGTAATCCGCATCCTGCAAGCGCGTATCTATGAGCAATTAACGATTGACCTCTGGGAAGACGCAGAAGACTTGCTCAATGAATACTTG‑‑‑‑‑‑‑‑‑G‑‑‑‑‑‑‑‑‑A‑‑‑‑‑‑‑‑‑GGAAGTCG‑‑‑‑‑‑‑C‑‑ACAACAGAAAGAAATTGACCGCGCTAAGGCCCGTAAAGAACGTCACGAGGGGCGCTTAGAGGCACGCAGATTCAAA‑CGTCGCAACCGCAAGGCACGTAAAGCACACAAAGCTAAGCGCGAAAGAATGCTTGC                                                                                                                                                                        <  1:134757/281‑1`  
                                                                                                                                              `aagagacagTGGCAAGTGAGGGCATTGACCTTGAGTTCGAAGACTCTGGTCTGATGCCTGACACCAAGGACGTAATCCGCATCCTGCAAGCGCGTATCTATGAGCAATTAACGATTGACCTCTGGGAAGACGCAGAAGACTTGCTCAATGAATACTTGGAGGAAGTCG‑‑‑‑‑‑‑‑‑A‑‑‑‑‑‑‑‑‑GGAAGTCG‑‑‑‑‑‑‑C‑‑ACGACAGAAAGAAATTGACCGCGCTAAGGCCCGTAAAGAACGTCATGAGGGGCGCTTAGATGCACGCAGATTCAAA‑CGTCGCAACCGCAAGGCACGTAAAGCACACAAAGCTAAGCGCGAAAG                                                                                                                                                                                 <  1:9518/292‑1`  
                                                                                                                                              `TTAGCCTAATGGCAAGTGAGGGCATTGACCTTGAGTTAGAAGACTCTGGTCTGATGCCTGACACCAAGGACGTAATCCGCATCCTGCAAGCGCGTATCTATGAGCAATTAACGATTGACCTCTGGGAAGACGCAGAAGACTTGCTCAATGAATACTTG‑‑‑‑‑‑‑‑‑G‑‑‑‑‑‑‑‑‑A‑‑‑‑‑‑‑‑‑GGAAGTCG‑‑‑‑‑‑‑C‑‑ACGACAGAAAGAAATTGACCGCGCTAAGGCCCGTAAAGAACGTCACGAGGGGCGCTTAGAGGCACGCAGATTCAAA‑CGTCGCAACCGCAAGGCACGTAAAGCACACAAAGCTAAGCGCGAAAGAATGCTTGC                                                                                                                                                                        <  1:189334/301‑1`  
                                                                                                                                                      `ATGGCAAGTGAGGGCATTGACCTTGAGTTCGAAGACTCTGGTCTGATGCCTGACACCAAGGACGTAATACGCATCCTGCAAGCGCGTATCTATGAGCAATTAACGATTGACCTCTGGGAAGACGCAGAAGACTTGCTCAATGAATACTTG‑‑‑‑‑‑‑‑‑G‑‑‑‑‑‑‑‑‑A‑‑‑‑‑‑‑‑‑GGAAGTCG‑‑‑‑‑‑‑C‑‑ACGACAGAAAGAAATTGACCGCGCTAAGGCCCGTAAAGAACGTCACGAGGGGCGCTTAGAGGCACGCAGATTCAAA‑CGTCGCAACCGCAAGGCACGTAAAGCACACAAAGCTAAGCGCGAAAGAATGCTTGCTGCGTGGC                                                                                                                                                                <  1:155918/301‑1`  
                                                                                                                                                       `gGGCAAGTGAGGGCATTGACCTTGAGTTCGAAGACTCTGGTCTGATGCCTGACACCAAGGACGTAATCCGCATCCTGCAAGCGCGTATCTATGAGCAATTAACGATTGACCTCTGGGAAGACGCAGAAGACTTGCTCAATGAATACTTGGAGGAAGTCG‑‑‑‑‑‑‑‑‑A‑‑‑‑‑‑‑‑‑GGAAGTCG‑‑‑‑‑‑‑C‑‑ACGACAGAAAGAAATTGACCGCGCTAAGGCCCGTAAAGAACGTCACGAGGGGCGCTTAGAGGCACGCAGATTCAAA‑CGTCGCAACCGCAAGGCACGTAAAGCACACAAAGCTAAGCGCGAAAGAATGCTTGC                                                                                                                                                                        <  1:48101/300‑1`  
                                                                                                                                                       `TGGCAAGTGAGGGCATTGACCTTGAGTTCGAAGACTCTGGTCTGATGCCTGACACCAAGGACGTAATCCGCATCCTGCAAGCGCGTATCTATGAGCAATTAACGATTGACCTCTGGGAAGACGCAGAAGACTTGCTCAATGAATACTTGGAGGAAGTCG‑‑‑‑‑‑‑‑‑A‑‑‑‑‑‑‑‑‑GGAAGTCG‑‑‑‑‑‑‑C‑‑ACGACAGAAAGAAATTGACCGCGCTAAGGCCCGTAAAGAACGTCACGAGGGGCGCTTAGAGGCACGCAGATTCAAC‑CGTCGCAACCGCAAGGCAAGTAAAGCACAACAAACTAAGCGCGAAAGAATGCTTGC                                                                                                                                                                        >  1:62571/1‑301`  
                                                                                                                                                          `aAAGTGAGGGCATTGACCTGGAGTTCGAAGACTCTGGGCTGATGCCTGACACCAAGGACGTAATCCGCATCCTGCAAGCGCGTATCTATGAGCAATTAACGATTGACCTCTGGGAAGACGCAGAAGACTTGCTCAATGAATACTTG‑‑‑‑‑‑‑‑‑G‑‑‑‑‑‑‑‑‑A‑‑‑‑‑‑‑‑‑GGAAGTCG‑‑‑‑‑‑‑C‑‑ACGACAGAAAGAAATTGACCGCGCTAAGGCCCGTAAAGAACGTCACGAGGGGCGCTTAGAGGCACGCAGATTCAAA‑CGTCGCAACCGCAAGGCACGTAAAGCACACAAAGCTAAGCGCGAAAGAATGCTTGCTGCGTGGCGATG                                                                                                                                                            <  1:294012/300‑1`  
                                                                                                                                                             `GTGCGGGCATTGACCTTGAGTTCGAAGACTCTGGTCTGATGCCTGACACCAAGGACGTAATCCGCATCCTGCAAGCGCGTATCTATGAGCAATTAACGATTGACCTCTGGGAAGATGCAGAAGACTCGCTCAATGAATACTTG‑‑‑‑‑‑‑‑‑G‑‑‑‑‑‑‑‑‑A‑‑‑‑‑‑‑‑‑GGAAGTCG‑‑‑‑‑‑‑C‑‑ACGACAGAACGAAATTGACCGCGCTAAGGCCCGTAAAGAACGTCACGAGGGGCGCTTAGAGGCACGCAGATTCAAA‑CGTCGCAACCGCAAGGCACGTAAAGCACACAAAGCTACGCGCGAAAGAATGCTTGCTGCGTGGCGATGGGC                                                                                                                                                         >  1:248112/1‑301`  
                                                                                                                                                             `GTGAGGGCATTGACCTTGAGTTCGAAGACTCTGGTCTGATGCCTGACACCAAGGACGTAATCCGCATCCTGCAAGCGCGTATCTATGAGCAATTAACGATTGACCTCTGGGAAGACGCAGAAGACTTACTCAATGAATACTTG‑‑‑‑‑‑‑‑‑T‑‑‑‑‑‑‑‑‑A‑‑‑‑‑‑‑‑‑GGAAGTCG‑‑‑‑‑‑‑C‑‑ACGACAGAAAGAAATTGACCGCGCTAAGGCCCGTAAAGAACGTCACGAGGGGCGCTTAGAGGCACGCAGATTCAAA‑CGTCGCAACCGCAAGGCACGTAAAGCACACCAAGCTAAGCGCGAAAGAATGCTTGCTGCGGTGCGATGGGg                                                                                                                                                         >  1:58795/1‑300`  
                                                                                                                                                             `GTGAGGGCAGTGACCTTGGGTTAGAAGACTCTGGTCTGATGCCTGACACCAAGGACGTAAGCCGCCTCCTGCAAGCGCGTATCTATGAGTAATTAACGATTGACCCCTGGGAAGACGCAGAAGACTTGCTCAATGAATACTTG‑‑‑‑‑‑‑‑‑G‑‑‑‑‑‑‑‑‑A‑‑‑‑‑‑‑‑‑GGAAGTCG‑‑‑‑‑‑‑C‑‑ACGACAGAAAGAAATTGACCGCGCTAAGGCCCGTAAAGAACGTCACGAGGGGCGCTTAGAGGCACGCAGATTCAAA‑CGTCGCAACCGCAAGGCACGTAAAGCACACAAAGCTAAGCGCGAAAGAATGCTTGCTGCGTGGCGATGGGC                                                                                                                                                         <  1:183053/301‑1`  
                                                                                                                                                                  `GGCATTGACCTTGAGTTCGAAGACTCTGGTCTGATGCCTGACACCAAGGGCGTAATCCGCATCCTGCAAGCGCGTATCTATGAGCAATTAACGATTGACCTCTGGGAAGACGCAGAAGACTTGCTCAATGAATACTTGGAGGAAGTCG‑‑‑‑‑‑‑‑‑A‑‑‑‑‑‑‑‑‑GGAAGTCG‑‑‑‑‑‑‑C‑‑ACGACAGAAAGAAATTGACCGCGCTAAGGCCCGTAAAGAACGTCACGAGGGGCGCTTAGAGGCACGCAGATTCAAA‑CGTCGCAACCGCAAGGCACGTAAAGCACACAAAGCTAAGCGCGAAAGAATGCTTGCTGCGTGGCGAT                                                                                                                                                             >  1:70140/1‑301`  
                                                                                                                                                                  `GGCATTGACCTTGAGTTCGAAGACTCTGGTCTGATGCCTGACACCAAGGACGTAATCCGCATCCTGCAAGCGCGTATCTATGAGCAATTAACGATTGACCTCTGGGAAGACGCAGAAGACTTGCTCAATGAATACTTGGAGGAAGTCG‑‑‑‑‑‑‑‑‑A‑‑‑‑‑‑‑‑‑GGAAGTCG‑‑‑‑‑‑‑C‑‑ACGACAGAAAGAAATTGACCGCGCTAAGGCCCGTAAAGAACGTCACTAGGGGCGCTTAGAGGCACGCAGATTCAAA‑CGTCGCAACCGCAAGGCACGTAAAGCACACAAAGCTAAGCGCGAAAGAATGCTTGCTGCGTGGCGAT                                                                                                                                                             >  1:303547/1‑301`  
                                                                                                                                                                        `GACCTTGAGTTCGAAGACTCTGGTCTGATGCCTGACACCAAGGACGTAATCCGCATCCTGCAAGCGCGTATCTATGAGCAATTAACGAATGACCTCTGGGAAGACGCAGAAGACTTGCTCAATGAATACTTGGAGGAAGTCG‑‑‑‑‑‑‑‑‑A‑‑‑‑‑‑‑‑‑GGAAGTCG‑‑‑‑‑‑‑C‑‑ACGACAGAAAGAAATTGACCGCGCTAAGGCCCGTAAAGAACGTCACGATGGGCGCTTAGAGGCACGCAGATTCAAA‑CGTCGCAACCGCAAGGCACGTAAAGCACACAAAGCTAAGCGCGAAAGAATGATTGCTGAGTGGCGAAGGGCTG                                                                                                                                                       >  1:174989/1‑301`  
                                                                                                                                                                         `ACCTTGAGTTCGAAGACTCTGGTCTGATGCCTGACACCAAGGACGTAATCCGCATCCTGCAAGCGCGTATCTTTGAGCAATTAACGATTGACCTCTGGGAAGACGCAGAAGACTTGCTCAATGAATACTTG‑‑‑‑‑‑‑‑‑G‑‑‑‑‑‑‑‑‑A‑‑‑‑‑‑‑‑‑GGAAGTCG‑‑‑‑‑‑‑C‑‑ACGACAGAAAGAAATTGACCGCGCTAAGGCCCGTAAAGAACGTCACGAGGGGCGCTTAGAGGCACTCAGATTCAAA‑CGTCGCAACCGCAAGTCACGTAAAGCACACAAAGCTAAGCGCGAAAGAATGCTTGCCGCGTGGCGACGGGCTGAACGTCAAGA                                                                                                                                             >  1:199132/1‑301`  
                                                                                                                                                                                `tTTCGAAGACCTTGGGCTGATTCCTGACACCAAGGACGTAATCCGCATCCTTCAAGCGCGTATCTATGAGCAATTAACGATTGACCTCTGGGAAGACGGAGAAGACTTGCTTAATGAATAATTG‑‑‑‑‑‑‑‑‑G‑‑‑‑‑‑‑‑‑A‑‑‑‑‑‑‑‑‑GGAAGTCG‑‑‑‑‑‑‑C‑‑ACGACAGAAAGAAATTGACCGCGCTAAGGCCCGTAAAGAACGTCACGAGGGGCGCTTAGAGGCACGCAGATTTAAA‑CGTCGCAACCGCAAGGCACGTAAAGCACACAAAGCTAAGCGCGAAAGAATGCTTGCTGCGTGGCGATGGGCTGAACGTCAAGAACGGCGT                                                                                                                                      <  1:153405/300‑1`  
                                                                                                                                                                                 `TTCGAAGACTCTGGTCTGATGCCTGACACCAAGGACGTAATCTGCATCCTGCAAGCGCGTATCTATGAGCAATCAACGATTGACCTCTGGGAAGACGCAGAAGACTTGCTCAATGAATACTTG‑‑‑‑‑‑‑‑‑GAGGAAGTCTA‑‑‑‑‑‑‑‑‑GGAAGTCG‑‑‑‑‑‑‑C‑‑ACGACAGAAAGAAATTGACCGCGCTAAGGCCCGTAAAGAACGTCACGAGGGGCGCTTAGAGGCACGCAGATTCAAA‑CGTCGCAACCGCAAGGCACGTAAAGCACACAAAGCTAACCGCGAAAGAATGCTTGCTGCGTGGCGATTGGCTGAACGTCAAG                                                                                                                                              >  1:181113/1‑301`  
                                                                                                                                                                                 `TTCGAAGACTCTGGTCTGATGCCTGACACCAAGGACGTAATCCGCATCCTGCAAGCGCGTATCTATGAGCAATTAACGATTGACCTCTGGGAAGACGCAGAAGACTTGCTCAATGAGTACTTG‑‑‑‑‑‑‑‑‑G‑‑‑‑‑‑‑‑‑A‑‑‑‑‑‑‑‑‑GGAAGTCG‑‑‑‑‑‑‑C‑‑ACGACAGAAAGAAATTGACCGCGCTAAGGCCCGTAAAGATCGTCACGAGGGGCGATTAGAGGCACGCAGATTCAAA‑CGTCGCAACCGCAAGGCACGTCAAGCACACCAAGCTAAACGAGAAAGAATGATTGCTGCGTGGCGATGGGTTGAACGCCAAAAACGGCGTA                                                                                                                                     >  1:207330/1‑301`  
                                                                                                                                                                                    `GAAGACTTTGGTATGTGGCCTGACAACAAGGACGTAATCCGCAGCCTACAAGAGAGTATCTATGAGCAATTAACGATTGACCTCTGGGAAGACGCAGAAGACTTGCTCAATGAATACTTGGAGGAAGTCG‑‑‑‑‑‑‑‑‑A‑‑‑‑‑‑‑‑‑GGAAGTCG‑‑‑‑‑‑‑C‑‑ACGACAGAAAGAAATTGACCGCGCTAAGGCCCGTAAAGAACGTCACGAGGGTCGCTTAGAGGCACGCAGATTCAAA‑CGTCGCAACCGCAAGGCACGTAAAACACACAAAGCTAAGCGCGAAAGAATGCTTGCTGCGTGGCGATGGGCTGAACGTCAAGAAC                                                                                                                                           <  1:256811/301‑1`  
                                                                                                                                                                                      `AGACTCTGGTCTGATGCCTGACACCAAGGACGTAATCCGCATCCTGCAAGCGCGTATCTATGAGCAATTAACGATTGACCTCTGGGAAGACGCAGAAGACTTGCTCAATGAATACTTG‑‑‑‑‑‑‑‑‑G‑‑‑‑‑‑‑‑‑A‑‑‑‑‑‑‑‑‑GGAAGTCG‑‑‑‑‑‑‑C‑‑ACGACAGAAAGAAATTGACCGCGCTAAGGCCCGTAAAGAACGTCACGAGGGGCGCTTAGAGGCACGCAGATTCAAA‑CGTCGCAACCGCAAGGCACGTAAAGCACACAAAGCTAAGCGCGAAAGAATGCTTGCTGCGTGGCGATGGGCTGAACGTCAAGAACGGCGTAAACAT                                                                                                                                <  1:12747/301‑1`  
                                                                                                                                                                                            `TGGTCTGATGCATGACACCAAGAACGTAATCCATATCCTGCAAGCGCGTATCTATGAGCAATTAACGATTGACCTCTGGGAAGACGCAGAAGACTTGCTCAATGAATACTTGGAGGAAGTCG‑‑‑‑‑‑‑‑‑A‑‑‑‑‑‑‑‑‑GGAAGTCG‑‑‑‑‑‑‑C‑‑ACGACAGAAAGAAATTGACCGCGCTAAGGCCCGTAAAGAACGTCACGAGGGGCGCTTAGAGGCACGCAGATTCAAA‑CGTCGCAACCGCAAGGCACGTAAAGCACACAAAGCTAAGCGCGAAAGAATGCTTGCTGCGTGGCGATGGGCTGAACGTCAAGAACGGCGTAAC                                                                                                                                   <  1:160748/301‑1`  
                                                                                                                                                                                              `GTCTGATGCCTGACACCAAGGACGTAATCCGCATCCTGCAAGCGCGTATCTATGAGCAATTAACGATTGACCTCTGGGAAGACGCAGAAGACTTGCTCAATGAATACTTGGAGGAAGTCG‑‑‑‑‑‑‑‑‑A‑‑‑‑‑‑‑‑‑GGAAGTCG‑‑‑‑‑‑‑C‑‑ACGACAGAAAGAAATTGACCGCGCTAAGGCCCGTAAAGAACGTCACGAGGGGCGCTTAGAGGCACGCAGATTCAAA‑CGTCGCAACCGCAAGGCACGTAAAGCACACAAAGCTAAGCGCGAAAGAATGCTTGCTGCGTGGCGACGGGCTGGACGTCAAGAACGGCGTAACCA                                                                                                                                 >  1:286097/1‑301`  
                                                                                                                                                                                                   `caGCCTGACACCAAGGACGTAATCCGCATCCTGCAAGCGCGTATCTATGAGCAATTAACGATTGACCTCTGGGAAGACGCAGAAGACTTGCTCAATGAATACTTGGAGGAAGTCG‑‑‑‑‑‑‑‑‑A‑‑‑‑‑‑‑‑‑GGAAGTCG‑‑‑‑‑‑‑C‑‑ACGACAGAAAGAAATTGACCGCGCTAAGGCCCGTAAAGAACGTCACGAGGGGCGCTTAGAGGCACGCAGATTCAAA‑CGTCGCAACCGCAAGGCACGTAAAGCACACAAAGCTAAGCGCGAAAGAATGCTTGCTGCGTGGCGATGGGCTGAACGTCAAGAACGGCGTAACCATGAGG                                                                                                                            <  1:262256/299‑1`  
                                                                                                                                                                                                   `aaGCCTGACACAAAGAACGTATTCCGCATCCTGAAAGCGCGTATCTATGAGCAATTAACGATTGACCGCTGGGAAGACGCAGAAGACTTGCTCAATGAATACTTG‑‑‑‑‑‑‑‑‑G‑‑‑‑‑‑‑‑‑A‑‑‑‑‑‑‑‑‑GGAAGTCGAGGAAGTCGAACGAAAGAAAGAAATTGACCGCGCTAAGGCCCGTAAAGAACGTCACGAGGGGCGCTTAGAGGCACGCAGATTCAAA‑CGTCGCAACCGCAAGGCACGTAAAGCACACAAAGCTAAGCGCGAAAGAATGCTTGCTGCGTGGCGATGGGCTGAACGTCAAGAACGGCGTAACCATGAGG                                                                                                                            <  1:335870/299‑1`  
                                                                                                                                                                                                     `acagGACACCAAGGACGTAATCCGCATCCTGCAAGCGCGTATCTATGAGCAATTAACGATTGACCTCTGGGAAGACGCAGAAGACTTGCTCAATGAATACTTG‑‑‑‑‑‑‑‑‑G‑‑‑‑‑‑‑‑‑A‑‑‑‑‑‑‑‑‑GGAAGTCG‑‑‑‑‑‑‑C‑‑ACGACAGAAAGAAATTGACCGCGCTAAGACCCGTAAAGAACGTCACGAGGGGCGCTTAGAGGCACGCAGATTCAAA‑CGTCGCAACCGCAAGGCACGTAAAGCACACAAAGCTAAGCGCGAAAGAATGCTTGCTGCGTGGCGATGGGCTGAACGTCAAGAACGGCGTAACCATGAGGTAGCTGTAGAT                                                                                                                 <  1:5028/297‑1`  
                                                                                                                                                                                                     `GCCTGACACCAAGGACGTAATCCGCATCCTGCAAGCGCGTATCTATTAGCAATTAACGATTGACCTCTGGGAAGACGCAGAAGACTTGCTCAATGAATACTTGGAGGAAGTCG‑‑‑‑‑‑‑‑‑A‑‑‑‑‑‑‑‑‑GGAAGTCG‑‑‑‑‑‑‑C‑‑ACGACAGAAAGAAATTGACCGCGCTAAGGCCCGTAAAGAACGTCACGAGGGGCGCTTAGAGGCACGCAGATTCAAA‑CGTCGCAACCGCAAGGCACGTAAAGCACACAAAGCTAAGCGCGAAAGAATGCTTGCTGCGTGGCGATGGGCTGAACGTCAAGAACGGCGTAACCATGAGGTA                                                                                                                          >  1:114002/1‑301`  
                                                                                                                                                                                                     `GCCTGACACCAAGGACGTAATCCGCATCCTGCAAGCGCGTATCTATGAGCAATTAACGATTGACCTCTGGGAAGACGCAGAAGACTTGCTCAATGAATACTTG‑‑‑‑‑‑‑‑‑G‑‑‑‑‑‑‑‑‑A‑‑‑‑‑‑‑‑‑GGAAGTCG‑‑‑‑‑‑‑C‑‑ACGACAGAAAGAAATTGACCGCGCTAAGGCCCGTAAAGAACGTCACGAGGGGCGCTTAGAGGCACGCAGATTCAAA‑CGTCGCAACCGCAAGGCACGTAAAGCACACAAAGCTAAGCGCGAAAGAATGCTTGCTGCGTGGCGATGGGCTGAACGTCAAGAACGGCGTAACCATGAGGTAGCTGTAGAT                                                                                                                 <  1:232891/301‑1`  
                                                                                                                                                                                                      `CCTGACACCAAGGACGTAATCCGCATCCTGCAAGCGCGTATCTATGAGCAATTAACGATTGACCTCTGGGAAGACGCAGAAGACTTGCTCAATGAATACTTG‑‑‑‑‑‑‑‑‑G‑‑‑‑‑‑‑‑‑A‑‑‑‑‑‑‑‑‑GGAAGTCG‑‑‑‑‑‑‑C‑‑ACGACAGAAAGAAATTGACCGCGCTAAGGCCCGTAAAGAACGTCACGAGGGGCGCTTAGAGGCACGCAGATTCAAA‑CGTCGCAACCGCAAGGCACGTCAAGCACACAAAGCTAAGCGCGAAAGAATGCTTGCTGCGTGGCGATGGGCTGAACGTCAAGAACGGCGTAACCATGAGGTAGCTGTAGATG                                                                                                                >  1:296682/1‑301`  
                                                                                                                                                                                                           `CACCAAGGACGTAATCCGCATCCTGCAAGCGCGTATCTATGAGCAATTAACGATTGACCTCTGGGAAGACGCAGAAGACTTGCTCAATGAATACTTG‑‑‑‑‑‑‑‑‑G‑‑‑‑‑‑‑‑‑A‑‑‑‑‑‑‑‑‑GGAAGTCG‑‑‑‑‑‑‑C‑‑ACGACAGAAAGAAATTGACCGCGCTAAGGCCCGTAAAGAACGTCACGAGGGGCGCTTAGAGGCACGCAGATTCAAA‑CGTCGCAACCGCAAGGCACGTAAAGCACACAAAGCTAAGCGCGAAAGAATGCTTGCTGCGTGGCGATGGGCTGAACGTCAAGAACGGCGTAACCATGAGGTAGCTGTAGAAGTACTA                                                                                                           >  1:172348/1‑301`  
                                                                                                                                                                                                             `CCAAGGACGTAATCCGCATCCTGCAAGCGCGTATCTATGAGCAATTAACGATTGACCTCTGGGAAGACGCAGAAGACTTGCTCAATGAATACTTGTAGGAAGTCG‑‑‑‑‑‑‑‑‑A‑‑‑‑‑‑‑‑‑GGAAGTCG‑‑‑‑‑‑‑C‑‑ACGACAGAAAGAAATTGACCGCGCTAAGGCCCGTAAAGAACGTCACGAGGGGCGCTTAGAGGCACGCAGATTCAAA‑CGTCGCAACCGCAAGGCACGTAAAGCACACAAAGCTAAGCGCGAAAGAATGCTTGCTGCGTGGCGATGGGCTGAACGTCAAGAACGGCGTAACCATGAGGTAGCTGTAGA                                                                                                                  <  1:320937/301‑1`  
                                                                                                                                                                                                              `ataagggacagTCCGCATCCTGCAAGCGCGTATCTATGAGAAATTAACGATTGACCTCTGGGAAGACGCAGAAGACTTGCTCAATGAATACTTG‑‑‑‑‑‑‑‑‑G‑‑‑‑‑‑‑‑‑A‑‑‑‑‑‑‑‑‑GGAAGTCG‑‑‑‑‑‑‑C‑‑ACGACAGAAAGAAATTGACCGCGCTAAGGCCCGTAAAGAACGTCACGAGGGGCGCTTAGAGGCACGCAGATTCAAA‑CGTCGCAACCGCAAGGCACGTAAAGCACACAAAGCTAAGCGCGAAAGAATGCTTGCTGCGTGGCGATGGGCTGAACGTCAAGAACGGCGTAACCATGAGGTAGCTGTAGATGTACTAGGA                                                                                                        <  1:271499/290‑1`  
                                                                                                                                                                                                              `ataagagacagTCCGCATCCTGCAAGCGCGTATCTATGAGAAATTAACGATTTACCTCTGGGAAGACGCAGAAGACTTGCTCAATGAATACTTG‑‑‑‑‑‑‑‑‑G‑‑‑‑‑‑‑‑‑A‑‑‑‑‑‑‑‑‑GGAAGTCG‑‑‑‑‑‑‑C‑‑ACGACAGAAAGAAATTGACCGCGCTAAGGCCCGTAAAGAACGTCACGAGGGGCGCTTAGAGGCACGCAGATTCAAA‑CGTCGCAACCGCAAGGCACGTAAAGCACACAAAGCTAAGCGCGAAAGAATGCTTGCTGCGTGGCGATGGGCTGAACGTCAAGAACGGCGTAACCATGAGGTAGCTGTAGATGTACTAGGA                                                                                                        <  1:250658/290‑1`  
                                                                                                                                                                                                              `aAAGGACGTAATCCGCATCCTGAAAGCGCGTATCTATGAGCAATTAACGATTGACCTCTGGGAAGACGCAGAAGACCTGCTCAATGAATACTTG‑‑‑‑‑‑‑‑‑G‑‑‑‑‑‑‑‑‑A‑‑‑‑‑‑‑‑‑GGAAGTCG‑‑‑‑‑‑‑C‑‑ACGACAGAAAGAAATTGACCGCGATAAGGCCCGTAAAGAACGTCACGAGGGGCGCTTAGAGGCACGCAGATTCAAA‑CGTCGCAACCGCAAGGCACGTAAAGCACACAAAGCTAAGCGCGAAAGAATGCTTGCTGCGTGGCGATGGGCTGAACGTCAAGAACGGCGTAACCATGAGGTAGCTGTAGATGTACTAGGA                                                                                                        <  1:10678/300‑1`  
                                                                                                                                                                                                                 `GGACGTAATCCGCATCCTGCAAGCGCGTATCTATGAGCAATTAACGATTGACCTCTGGGAAGACGCAGAAGACTTGCTCAATGAATACTTGGAGGAAGTCG‑‑‑‑‑‑‑‑‑A‑‑‑‑‑‑‑‑‑GGAAGTCG‑‑‑‑‑‑‑C‑‑ACGACAGGAAGAAATTGACCGCGCTAAGGCCCGTAAAGAACGTCACGAGGGGCGCTTAGAGGCACGCAGATTCAAA‑CGTCGCAACCGCAAGGCACGTAAAGCACACAAAGCTAAGCGCGAAAGAATGATTGCTGCGTGGCGATGGGCTGACCGTCAAGAACGGCGTAACCATGAGGGCGCTGTTGATGTA                                                                                                              >  1:122058/1‑301`  
                                                                                                                                                                                                                  `GACGTAAGCCGCATCCTGCAAGCGCGTATCTATGAGCAATTAACGATTGACCTCTGGGAAGACGCAGAAGACTTGCTCAATGAATACTTG‑‑‑‑‑‑‑‑‑G‑‑‑‑‑‑‑‑‑A‑‑‑‑‑‑‑‑‑GGAAGTCGAGGAAGTCGAACGACAGAAAGAAATTGACCGCGCTAAGGCCCGTAAAGAACGTCACGATGGGCGCTTAGAGGCACGCAGATTCAAA‑CGTCGCAACCGCAAGTCACGTAAAGCACACAAAGCTAAGCGCGAAAGAATGCTTGCTGCGTGGCGATGGGCTGAACGTCAAGAACGGCGTAACCATGAGGTAGCTGTAGATGTAC                                                                                                             <  1:202521/301‑1`  
                                                                                                                                                                                                                       `AATCTGCATCCTGCAAGCGCGTATCTATGAGCAATTAACGATTGACCTCTGGGAAGACGCAGAAGACTTGCTCAATGAATACTTGGAGGAAGTCG‑‑‑‑‑‑‑‑‑A‑‑‑‑‑‑‑‑‑GGAAGTCG‑‑‑‑‑‑‑C‑‑ACGACAGAAAGAAATTGACCGCGCTAAGGCCCGTAAAGAACGTCACGAGGGGCGCTTAGAGGCACGCAGATTCAAA‑CGTCGCAACCGCAAGGCACGTAAAGCACACAAAGCTAAGCGCGAAAGAATGCTTGCTGCGTGGCGATGGGCTGAACGTCAAGAACGGCGTAACCATGAGGTAGCTGTAGATGTACTAGGA                                                                                                        >  1:215912/1‑301`  
                                                                                                                                                                                                                       `AATCCGCATACTGCAAGGGGGTATCTATTAGCAATTAACGATTGACATCTGGGAAGACGCAGAAGACTTGCTAAATGAATACTTGGAGGAAGTCG‑‑‑‑‑‑‑‑‑A‑‑‑‑‑‑‑‑‑GGAAGTCG‑‑‑‑‑‑‑C‑‑ACGACAGAAAGAAATTGACCGCGCTAAGGCCCGGAAAGAACGTCACGAGGGGCGCTTAGAGGCACGCAGATTCAAA‑CGTCGCAACCGCAAGGCACGTAAAGCACACAAAGCTAAGCGCGAAAGAATGCTTGCTGCGTGGCGATGGGCTGAACGTCAAGAACGGCGTAACCATGATGTAGCTGTAGATGTACTAGGA                                                                                                        <  1:338481/301‑1`  
                                                                                                                                                                                                                               `TCCTGCAAGCGCGTATCTATGAGCAATTAACGATTGACCTCTGGGAAGACGCAGAAGACTTGCTCAATGCATACTTG‑‑‑‑‑‑‑‑‑G‑‑‑‑‑‑‑‑‑A‑‑‑‑‑‑‑‑‑GGAAGTCG‑‑‑‑‑‑‑C‑‑ACGACAGAAAGAAATTGACCGCGCTAAGGCCCGTAAAGAACGTCACGAGGGGCGCTTAGAGGCACGCAGATTCAAA‑CGTCGCAACCGCAAGGCACGTAAAGCACACAAAGCTAAGCGCGAAAGAATGCTTGCTGCGTGGCGATGGGCTGAACGTCAAGAACGGCGTAACCATGAGGTAGCTGTAGATGctgtctcttatacacatctccgagc                                                                                       >  1:147008/1‑276`  
                                                                                                                                                                                                                               `TCCTGCAAGCGCGTATCTATGAGCAATTAACGATTGACCTCTGGGAAGACGCAGAAGACTTGCTCAATGAATACTTGGAGGAAGTCG‑‑‑‑‑‑‑‑‑A‑‑‑‑‑‑‑‑‑GGAAGTCG‑‑‑‑‑‑‑C‑‑ACGACAGAAAGAAATTGACCGCGCTAAGGCCCGTAAAGAACGTCACGAGGGGCGCTTAGAGGCACGCAGATTCAAA‑CGTCGCAACCGCAAGGTACGTAAAGCACACAAAGCTAAGCGCGAAAGAATGCTTGCTGCGTGGCGATTGGCTGAACGTCAAGAACGGCGTAACCATGAGGTAGCTGTAGATGTACctgtctcttatac                                                                                                >  1:245308/1‑288`  
                                                                                                                                                                                                                               `TCCTGCAAGCGCGTATCTATGAGCAATTAACGATTGACCTCTGGGAAGACGCAGAAGACTTGCTCAATGAATACTTG‑‑‑‑‑‑‑‑‑G‑‑‑‑‑‑‑‑‑A‑‑‑‑‑‑‑‑‑GGAAGTCG‑‑‑‑‑‑‑C‑‑ACGACAGAAAGAAATTGACCGCGCTAAGGCCCGTAAAGAACGTCACGAGGGGCGCTTAGAGGCACGCAGATTCAAA‑CGTCGCAACCGCAAGGCACGTAAAGCACACAAAGCTAAGCGCGAAAGAATGCTTGCTGCGTGGCGATGGGCTGAACGTCAAGAACGGCGTAACCATGAGGTAGCTTTAGATGTACTAGGActgtctcttatacacat                                                                                       >  1:324747/1‑284`  
                                                                                                                                                                                                                                 `CTGCAAGCGCGTATCTATGAGCAATTAACGATTGACCTCTGGGAAGACGCAGAAGACTTGCTCAATGAATACTTG‑‑‑‑‑‑‑‑‑G‑‑‑‑‑‑‑‑‑A‑‑‑‑‑‑‑‑‑GGAAGTCG‑‑‑‑‑‑‑C‑‑ACGACAGAAAGAAATTGACCGCGCTAAGGCCCGTAAAGAACGTCACGAGGGGCGCTTAGAGGCACGCAGATTCAAA‑CGTCGCAACCGCAAGGCACGTAAAGCACACAAAGCTAAGCGCGAAAGAATGCTTGCTGCGTGGCGATGGGCTGAACGTCAAGAACGGCGTAACCATGAGGTAGCTGTAGATGTACTAGGAAGAACCAATAACGCTATGC                                                                                     >  1:186621/1‑301`  
                                                                                                                                                                                                                                 `CTGCAAGCGCGTATCTATGAGCAATTAACGATTGACCTCTGGGAAGACGCAGAAGACTTGCTCAATGAATACTTG‑‑‑‑‑‑‑‑‑G‑‑‑‑‑‑‑‑‑A‑‑‑‑‑‑‑‑‑GGAAGTCG‑‑‑‑‑‑‑C‑‑ACGACAGAAAGAAATTGACCGCGCTAAGGCCCGTAAAGAACGTCACGAGGGGCGCTTAGAGGCACGCAGATTCAAA‑CGTCGCAACCGCAAGGCACGTAAAGCACACAAAGCTAAGCGCGAAAGAATGCTTGCTGCGTGGCGAGGGGCTGAACGTCAAGAACGGCGTAACCATGGGGTAGCTGTAGAGGTACTAGGAAGACCCAATAACGCTATGC                                                                                     >  1:268915/1‑301`  
                                                                                                                                                                                                                                 `CTGCAAGCGCGTATCTATGAGCAATTAACGATTGACCTCTGGGAAGACGCAGAAGACTTGCTCAATGAATACTTG‑‑‑‑‑‑‑‑‑G‑‑‑‑‑‑‑‑‑A‑‑‑‑‑‑‑‑‑GGAAGTCG‑‑‑‑‑‑‑C‑‑ACGACAGAAAGAAATTGACCGAGCTAAGGCCCGTAAAGAACGTCACGAGGGGCGCTTAGAGGCACGCAGATTAAAA‑CGTCGCAACCGCAAGGCACGTAAAGCACACAAAGCTAAGCGCGAAAGAATGCTTGCTGCGTGGCGATGGGCTGAACGTCAAGAACGGCGTAACCATGAGGTAGCTGTAGATGTACTAGGAAGAACCAATAACtgtctct                                                                                     >  1:115608/1‑294`  
                                                                                                                                                                                                                                   `GCAAGCGGGTATCTATGAGAAATTAAAGATTGACCTCTGGGAAGACGCAGAAGACTTGCTCAATGAATACTTG‑‑‑‑‑‑‑‑‑G‑‑‑‑‑‑‑‑‑A‑‑‑‑‑‑‑‑‑GGAAGTCG‑‑‑‑‑‑‑C‑‑ACGAAAGAAAGAAATTGACCGCGCTAAGGCCCGTAAAGAACGTCACGAGGGGCGCTTAGAGGCACGCAGATTCAAA‑CGTCGCAACCGCAAGGCACGTAAAGCACACAAAGCTAAGCGCGAAAGAATGCTTGCTGCGTGGCGATGGGCTGAACGTCAAGAACGGCGTAACCATGAGGTAGCTGTAGATGTACTAGGAAGAACCAATAACGCTATGCTC                                                                                   <  1:67187/301‑1`  
                                                                                                                                                                                                                                    `ccAGCGCGTATCTATGAGCAATTAACGATTGACCTCTGGGAAGACGCAGAAGACTTGCTCAATGAATACTTG‑‑‑‑‑‑‑‑‑G‑‑‑‑‑‑‑‑‑A‑‑‑‑‑‑‑‑‑GGAAGTCG‑‑‑‑‑‑‑C‑‑ACGACAGAAAGAAATTGACCGCGCTAAGGCCCGTAAAGAACGTCACGAGGGGCGCTTAGAGGCACGCAGATTCAAACCGTCGCAACCGCAAGGCACGTAAAGCACACAAAGCTAAGCGCGAAAGAATGCTTGCTGCGTGGCGATGGGCTGAACGTCAAGAACGGCGTAACCATGAGGTAGCTGTAGATGTACTAGGAAGAACAAATAACGCTATGCTC                                                                                   <  1:36266/299‑1`  
                                                                                                                                                                                                                                     `AAGCGCGTATCTATGAGCAATTAACGATTGACCTCTGGGAAGACGCAGAAGACTTGCTCAATGAATACTTGGAGGAAGTCG‑‑‑‑‑‑‑‑‑A‑‑‑‑‑‑‑‑‑GGAAGTCG‑‑‑‑‑‑‑C‑‑ACGACAGAAAGAAATTGACCGCGCTAAGGCCCGTAAAGAACGTCACGAGGGGCGCTTAGAGGCACGCAGATTCAAA‑CGTCGCAACCGCAAGGCACGTAAAGCACACAAAGCTAAGCGCGAAAGAATGCTTGCTGCGTGGCGATGGGCTGAACGTCAAGAACGGCGTAACCATGAGGTAGCTGTAGATGTACTAGGAAGAACCAATAACGC                                                                                          <  1:77820/301‑1`  
                                                                                                                                                                                                                                            `TATCTATGAGCAATTAACGATTGACCTCTGGGAAGACGCAGAAGACTTGCTCAATGAATACTTGGAGGAAGTCG‑‑‑‑‑‑‑‑‑A‑‑‑‑‑‑‑‑‑GGAAGTCG‑‑‑‑‑‑‑C‑‑ACGACAGAAAGAAATTGACCGCGCTAAGGCCCGTAAAGAACGTCACGAGGGGCTCTTAGAGGCACGCAGATTCAAA‑CGTCGCAACCGCAAGGCACGTAAAGCACACAAAGCTAAGCGCGAAAGAATGCTTGCTGCGTGGCGATGTGCTGAACGTCAAGAACGGCGTAACCATGAGGTAGCTGTAGATGTACTAGGAAGAACCAATAACGCTATGCTC                                                                                   >  1:91532/1‑301`  
                                                                                                                                                                                                                                             `gTCTATGAGCAATTAACGATTGACCTCTGGGAAGACGCAGAAGACTTGCTCAATGAATACTTG‑‑‑‑‑‑‑‑‑G‑‑‑‑‑‑‑‑‑A‑‑‑‑‑‑‑‑‑GGAAGTCG‑‑‑‑‑‑‑C‑‑ACGACAGAAAGAAATTGACCGCGCTAAGGCCCGTAAAGAACGTCACGAGGGGCGCTTAGAGGCACGCAGATTCAAA‑CGTCGCAACCGCAAGGCACGTAAAGCACACAAAGCTAAGCGCGAAAGAATGCTTGCTGCGTGGCGATGGGCTGAACGTCAAGAACGTCGTAACCATGAGGTAGCTGTAGATGTACTAGGAAGAACCAATAACGCTATGCTCTGGGTCAACA                                                                         <  1:278054/300‑1`  
                                                                                                                                                                                                                                                `gagatgtgtataagagacagCCTCTGGGAAGACGCAGAAGACTTGCTCAATGAATACTTG‑‑‑‑‑‑‑‑‑G‑‑‑‑‑‑‑‑‑A‑‑‑‑‑‑‑‑‑GGAAGTCG‑‑‑‑‑‑‑C‑‑ACGACAGAAAGAAATTGACCGCGCTAAGGCCCGTAAAGAACGTCACGAGGGGCGCTTAGAGGCACGCAGATTCAAA‑CGTCGCAACCGCAAGGCACGTAAAGCACACAAAGCTAAGCGCGAAAGAATGCTTGCTGCGTGGCGATGGGCTGAACGTCAAGAACGGCGTAACCATGAGGTAGCTGTAGATGTACTAGGAAGAACCAATAACGCTATGCTCTGGGTCAACATGT                                                                      <  1:235820/281‑1`  
                                                                                                                                                                                                                                                    `cgaAATTAACGATTTACTTCTGGGAAGACGCAGAAGACTTGCTCAATGAATACTTG‑‑‑‑‑‑‑‑‑G‑‑‑‑‑‑‑‑‑A‑‑‑‑‑‑‑‑‑GGAAGTCG‑‑‑‑‑‑‑C‑‑ACGACAGAAAGATATTGACCGCGCTAAGGCCCGTAAAGAACGTCACGAGGGGCGCTTAGAGGCACGCAGATTCAAA‑CGTCGCAACCGCAAGGCACGTAAAGCACACAAAGCTAAGCGCGAAAGAATGCTTGCTGCGTGGCGATGGGCTGAACGTCAATAACTGCGTAACCATGAGGTAGCTGTAGATGTACTAGGAAGAACCAATAACGCTATGCTCTGGGTCAACATGTTCTC                                                                  <  1:100236/298‑1`  
                                                                                                                                                                                                                                                      `CAATTAACGATTGACCTCTGGGAAGACGCAGAAGACTTGCTCAATGAATACTTG‑‑‑‑‑‑‑‑‑G‑‑‑‑‑‑‑‑‑A‑‑‑‑‑‑‑‑‑GGAAGTCG‑‑‑‑‑‑‑C‑‑ACGACAGAAAGAAATTGACCGCGCTAAGGCCCGTAAAGAACGTCACGAGGGGCGCTTAGAGGCACGCAGATTCAAA‑CGTCGCAACCGCAAGGCACGTAAAGCACACAAAGCTAAGCGCGAAAGAATGCTTGCTGCGTGGCGATGGGCTGAACGTCAAGAACGGCGTAACCATGAGGTAGCTGTAGATGTACTAGGAAGCACCAATAACGCTATGCACTGGGTCAACATGTACTCTG                                                                >  1:225029/1‑301`  
                                                                                                                                                                                                                                                       `ggcgaggagatgtgtataagagacaaGCAGAAGACTTGCACAATGAATACTTG‑‑‑‑‑‑‑‑‑G‑‑‑‑‑‑‑‑‑A‑‑‑‑‑‑‑‑‑GGAAGTCG‑‑‑‑‑‑‑A‑‑ACGACATAAATAAATTGACCGCGCTAAGGCCCGTAAAGAACGTAAAGAGGGGCACGTAGAGGAACGCAGATTAAAA‑CGTCGCAAACGCAAGGCACGTAAAGCACACAAAGCTAAGCGCGAAAGAATGCTTGCTGCGTGGCGATGGGCTGAACGTCGAGAACGGAGTAACCATGAGGGAGCTGTAGATGTACGAGGAAGAACCAATAACGCTATGCTCTGGGTCAACATGTTCTCTGG                                                               <  1:341639/275‑1`  
                                                                                                                                                                                                                                                        `ATTAACGATTGACCTCTGGGAAGACGCAGAAGACTTGCTCAATGAATACTTGGAGGAAGTCG‑‑‑‑‑‑‑‑‑A‑‑‑‑‑‑‑‑‑GGAAGTCG‑‑‑‑‑‑‑C‑‑ACGACAGAAAGAAATTGACCGCGCTAAGGCCCGTAAAGAACGTCACGAGGGGCGCTTAGAGGCACGCAGATTCAAA‑CGTCGCAACCGCAAGGCACGTAAAGCACACAAAGCTAAGCGCGAAAGAATGCTTGCTGCGTGGCGATGGGCTGAACGTCAAGAACGGCGTAACCATGAGGTAGCTGTAGATGTACTAGGAAGAACCAATAACGCTATGCTCTGGGTCAACATG                                                                       >  1:273845/1‑301`  
                                                                                                                                                                                                                                                        `ATTAACGATTGACCTCTGGGAAGACGCAGAAGACTTGCTCAATGAATACTTG‑‑‑‑‑‑‑‑‑T‑‑‑‑‑‑‑‑‑A‑‑‑‑‑‑‑‑‑TGAAGTCG‑‑‑‑‑‑‑C‑‑ACGACAGAAAGAAATTGACCGCGCTAAGGCCCGTAAAGAACGTCACGAGGGGCGCTTAGAGGCACGCAGATTCAAA‑CGTCGCAACCGCAAGGCACGTAAAGCACACAAAGCTAAGCGCGAAAGAATGCTTGCTGCGTGGCGATGGGCTGAACGTCAAGAACGGCGGAACCATGAGGTAGATGTAGATGTACTAGGAAGAACCAATAACGCTATGCTCTGGGTCAACATGTTATCctgt                                                              >  1:194955/1‑297`  
                                                                                                                                                                                                                                                             `cgtTTGACCTATTGGAAGACGCAGAAGACTTGCTCAATGAAGACTTGGAGGAAGGCG‑‑‑‑‑‑‑‑‑A‑‑‑‑‑‑‑‑‑GGAAGTCG‑‑‑‑‑‑‑C‑‑ACGACAGAAAGAAATTGACCGCGCTAAGGCCCGTAAAGAACGTCACGAGGGGCGCTTAGAGGCACGCAGAATCAAA‑CGTCGCAACCGCAAGGCACGTAAAGCACACAAAGCTAAGCGCGAAAGAATGCTTGCTGCGTGGCGATGGGCTGAACGTCAAGAACGGCGTAACCATGAGGTAGCTGTAGATGTACTAGGAAGAACCAATAACGCTATGCTCTGGGTCAACATGTTCTC                                                                  <  1:308841/298‑1`  
                                                                                                                                                                                                                                                                      `cCTGGGAAGACGCAGAAGACTTGCTCAATGATTACTTG‑‑‑‑‑‑‑‑‑G‑‑‑‑‑‑‑‑‑A‑‑‑‑‑‑‑‑‑GGAAGTCG‑‑‑‑‑‑‑C‑‑ACGACAGAAAGAAATTGACCGCGCTAAGGCCCGTAAAGAACGTCACGAGGGGCGCTTAGAGGCACGCAGATTCAAA‑CGTCGCAACCGCAAGGCACGTAAAGCACACAAAGCTAAGCGCGAAAGAATGCTTGCTGCGTGGCGATGGGCTGAACGTCAAGAACGGCGTAACCATGAGGTAGCTGTAGATGTACTAGGAAGAACCAATAACGCTATGCTCTGGGTCAACATGTTCTCTGGGGACTTTAAGGCGCT                                                <  1:118891/300‑1`  
                                                                                                                                                                                                                                                                           `acAGACGCAGAAGACTTGCTCAATGAATACTTGGAGGAAGTCG‑‑‑‑‑‑‑‑‑A‑‑‑‑‑‑‑‑‑GGAAGTCG‑‑‑‑‑‑‑C‑‑ACGACAGAAAGAAATTGACCGCGCTAAGGCCCGTAAAGAACGTCACGAGGGGCGCTTAGAGGCACGCAGATTCAAA‑CGTCGCAACCGCAAGGCACGTAAAGCACACAAAGCTAAGCGCGAAAGAATGCTTGCTGCGTGGCGATGGGCTGAACGTCAAGAACGGCGTAACCATGAGGTAGCTGTAGATGTACTAGGAAGAACCAATAACGCTATGCTCTGGGTCAACATGTTCTCTGGGGACTTTAAGG                                                    <  1:253794/299‑1`  
                                                                                                                                                                                                                                                                             `AGACGCAGAAGACTTGCTCAATGAATACTTG‑‑‑‑‑‑‑‑‑G‑‑‑‑‑‑‑‑‑A‑‑‑‑‑‑‑‑‑GGCAGTCG‑‑‑‑‑‑‑C‑‑ACGACAGAAAGAAATTGACCGCGCTAAGTCCCGTACAGAACGTCACGAGGGGCGCTTAGAGGCACGCAGATTCAAA‑CGTCGCAACCGCAAGGCACGTAAAGCACACAAAGCTAAGCGCGAAAGAATGCTTGCTGCGTTGCGATGGGCTGAACGTCAAGAACGACGTAACCATGAGGTAGCTTTAGATGTACTAGGAAGAACCAATAACGCTATGCTCTGGGTCAACATGTTCTCTGGGGACTTTAAGGCGCTTGAGGAA                                         <  1:168398/301‑1`  
                                                                                                                                                                                                                                                                              `GACGCAGAAGACTTGCTCAATGAATACTTGGAGGAAGTCG‑‑‑‑‑‑‑‑‑A‑‑‑‑‑‑‑‑‑GGAAGTCG‑‑‑‑‑‑‑C‑‑ACGACAGAAAGAAATTGACCGCGCTAAGGCCCGTAAAGAACGTCACGAGGGGCGCTTAGAGGCACGCAGATTCAAA‑CGTCGCAACCGCAAGGCACGTAAAGCACACAAAGCTAAGCGCGAAAGAATGCTTGCTGCGTGGCGATGGGCTGAACGTCAAGAACGGCGTAAACATGAGGTAGCTGTAGATGTACTAGGAAGAACCAATAACGATATGCTCTGGGTCAACATGTTCTCTGGGGACTTTAAGGCGg                                                 >  1:315223/1‑300`  
                                                                                                                                                                                                                                                                                       `GACTTGCTCAATGAATACTTG‑‑‑‑‑‑‑‑‑G‑‑‑‑‑‑‑‑‑A‑‑‑‑‑‑‑‑‑GGAAGTCG‑‑‑‑‑‑‑C‑‑ACGACAGAAAGAAATTGACCGCGCTAAGGCCCGTAAAGAACGTCACGAGGGGCGCTTAGAGGCACGCAGATTCAAA‑CGTCGCAACCGCAAGGCACGTAAAGCACACAAAGCTAAGCGCGAAAGAATGCTTGCTGCGTGGCGATGGGCTGAACGTCAAGAACGGCGTAACCATGAGGTAGCTGTAGATGTACTAGGAAGAACCAATAACGCTATGCTCTGGGTAAACATGTTCTCTGGGGACTTTAAGGCGCTTGAGGAACGAATCGCGC                               <  1:55927/301‑1`  
                                                                                                                                                                                                                                                                                               `acagGAATACTAG‑‑‑‑‑‑‑‑‑G‑‑‑‑‑‑‑‑‑A‑‑‑‑‑‑‑‑‑GGAAGTCG‑‑‑‑‑‑‑C‑‑ACGACAGAAAGAAATTGACCGCGCTAAGGCCCGTAAAGAACGACACGAGGGGCGCATAGAGGCACGCAGATTCAAA‑CGTCGCAACCGCAAGGCACGTAAAGCACACAAAGCTAAGCGAGAAAGAATGCTTGCTGCGTGGCGATGGGCTGAACGTCAAGAACGGCGTAACAATGAGGTAGCTGTAGATGTACTAGGAAGAACCAATAACGCTATGCTCTGGGTCAACATGTTCTCTGGGGACTTTAAGGCGATTGAGGAACGAATCGCGCTGCACTGG                       <  1:210897/297‑1`  
                                                                                                                                                                                                                                                                                                 `ATGAATACGTG‑‑‑‑‑‑‑‑‑G‑‑‑‑‑‑‑‑‑A‑‑‑‑‑‑‑‑‑GGAAGTCG‑‑‑‑‑‑‑C‑‑ACGAAAGAAAGAAATTGACCGCGCTAAGGCCCGTAAAGAACGTCACGAGGGGCGCTTAGAGGCACGCAGATTCAAA‑CGTCGCAACCGCAAGGCACGTAAAGCACACAAAGCTAAGCGCGAAAGAATGCTTGCTGCGTGGCGATGGGCTGAACGTCAAGAACGGCGTAACCATGAGGTAGCTGTAGATGTACTAGGAAGAACCAATAACGCTATGCTCTGGGTCAACATGTTCTCTGGGGACTTTAAGGCGCTTGAGGAACGAATCGCGCTGCACTGGCG                     <  1:213412/301‑1`  
                                                                                                                                                                                                                                                                                                      `aACTTG‑‑‑‑‑‑‑‑‑G‑‑‑‑‑‑‑‑‑A‑‑‑‑‑‑‑‑‑GGAAGTCG‑‑‑‑‑‑‑A‑‑ACGAAAGAAAGAAATTGACCGCGCTAAGGCCCGTAAAGAACGTCACGAGGGGCGCTTAGAGGCACGCAGATTCAAA‑CGTCGCAACCGCAAGGCACGTAAAGCACACAAAGCTAAGCGCGAAAGAATGCTTGCTGCGTGGCGATGGGCTGAACGTCAAGAACGGCGTAACCATGAGGTAGCTGTAGATGTACTAGGAAGAACCAATAACGCTATGCTCTGGGTCAACATGTTCTCTGGGGACTTTAAGGCGCTTGAGGAACGAATCGCGCTGCACTGGCGTAATG                <  1:191955/300‑1`  
                                                                                                                                                                                                                                                                                                      `TACTTG‑‑‑‑‑‑‑‑‑G‑‑‑‑‑‑‑‑‑A‑‑‑‑‑‑‑‑‑GGAAGTCG‑‑‑‑‑‑‑C‑‑ACGACAGAAAGAAATTGACCGCGCTAAGGCCCGTAAAGAACGTCACGAGGGGAGCTTAGAGGCACGCAGATTCAAA‑CGTCGCAACCGCAAGGCACGTAAAGCACACAAAGCTAAGCGCGAAAGAATGCTTGCTGCGTGGCGATGGGCTGAACGTCAAGCACGGCGTAACCATGAGGTAGCTGTAGATGTACTAGGAAGAACCAATAACGCTATGCTCTGGGTCAACATGTTCTCTGGGGACTTTAAGGCGCTTGAGGAACGAATCGCGCTGCACTGGCGTAATt                >  1:89711/1‑300`  
                                                                                                                                                                                                                                                                                                        `CTTG‑‑‑‑‑‑‑‑‑G‑‑‑‑‑‑‑‑‑A‑‑‑‑‑‑‑‑‑GGAAGTCG‑‑‑‑‑‑‑C‑‑ACGACAGAAAGAAATTGACCGCGCTAAGGCCCGTAAAGAACGTCACGAGGGGCGCTTAGAGGCACGCAGATTCAAA‑CGTCGCAACCGCAAGGCACGTAAAGCACACAAAGCTAAGCGCGAAAGAATGCTTGCTGCGTGGCGATGGGCTGAACGTCAAGAACGGCGTAACCATGAGGTAGCTGTAGATGTACTAGGAAGAACCAATAACGCTATGCTCTGGGTCAACATGTTCTCTGGGGACTTTAAGGCGCTTGAGGAACGAATCtgtctcttatacacatctccc              >  1:312453/1‑280`  
                                                                                                                                                                                                                                                                                                           `G‑‑‑‑‑‑‑‑‑G‑‑‑‑‑‑‑‑‑A‑‑‑‑‑‑‑‑‑GGAAGTCG‑‑‑‑‑‑‑C‑‑ACGACAGAAAGAAATTGACCGCGCTAAGGCCCGTAAAGAACGTCACGAGGGGCGCTTAGAGGCACGCAGATTCAAA‑CGTCGCAACCGCAAGGCACGTAAAGCACACAAAGCTAAGCGCGAAAGAATGCTTGCTGCGTGGCGATGGGCTGAACGTCAAGAACGGCGTAACCATGAGGTAGCTGTAGATGTACTAGGAAGAACCAATAACGCTATGCTCTGGGTCAACATGTTCTCTGGGGACTTTAAGGCGCTTGAGGAACGAATCGCGCTGCACTGGCGTAATGCTGAC           >  1:41752/1‑301`  
                                                                                                                                                                                                                                                                                                                     `G‑‑‑‑‑‑‑‑‑A‑‑‑‑‑‑‑‑‑GGAAGTCG‑‑‑‑‑‑‑C‑‑ACGACAGAAAGAAATTGACCGCGCTAAGGCCCGTAAAGAACATCACTAGGGGCGCTTAGAGGCACGCAGATTCAAA‑CGTCGCAACCGCAAGGCACGTAAAGCACACAAAGCTAAGCGCGAAAGAATGCTTGCTGCGTGGCGATGGGCTGAACGTCAAGAACGGCGTAACCATGAGGTAGCTGTAGATGTACTAGGAAGAACCAATAACGCTATGCTCTGGGTCAACATGTTCTCTGGGGACTTTAAGGCGCTTGAGGAACGAATCGCGCTGCACTGGCGTAATGCTGACC          <  1:158801/301‑1`  

                                                                                                                                                                                                                                                                                                                                                                                                                                                                                                                                                                                                                                                                        
`ACTTACAACAACGTTTTCGACCACGCTTACGAAATGCTGAAAGAAAACATCCGTTATGATGACATCCGTGACACTGATGACCTGCACGATGCTATTCACATGGCTGCCGATAATGCAGTTCCGCACTACTACGCTGACATCTTTAGCGTAATGGCAAGTGAGGGCATTGACCTTGAGTTCGAAGACTCTGGTCTGATGCCTGACACCAAGGACGTAATCCGCATCCTGCAAGCGCGTATCTATGAGCAATTAACGATTGACCTCTGGGAAGACGCAGAAGACTTGCTCAATGAATACTTG‑‑‑‑‑‑‑‑‑G‑‑‑‑‑‑‑‑‑A‑‑‑‑‑‑‑‑‑GGAAGTCG‑‑‑‑‑‑‑‑‑‑‑‑‑‑‑‑‑‑‑‑‑‑‑‑‑‑‑‑‑‑‑‑‑‑‑‑‑‑‑‑‑‑‑‑‑‑‑‑‑‑‑‑‑‑‑‑‑‑‑‑‑‑‑‑‑‑‑‑‑‑‑‑‑‑‑‑‑‑‑‑‑‑‑‑‑‑‑‑‑‑‑‑‑‑‑‑‑‑‑‑‑‑‑‑‑‑‑‑‑‑‑‑‑‑‑‑‑‑‑‑‑‑‑‑‑‑‑‑‑‑‑‑‑‑‑‑‑‑‑‑‑‑‑‑‑‑‑‑‑‑‑‑‑‑‑‑‑‑‑‑‑‑‑‑‑‑‑‑‑‑‑‑‑‑‑‑‑‑‑‑‑‑‑‑‑‑‑‑‑‑‑‑‑‑‑‑‑‑‑‑‑‑‑‑‑‑‑‑‑‑‑‑‑‑‑‑‑‑‑‑‑‑‑‑‑‑‑‑‑‑‑‑‑‑‑‑‑‑‑‑‑‑‑‑‑‑‑‑‑‑‑‑‑‑‑‑‑‑‑‑‑‑‑‑‑‑‑‑‑‑‑‑‑‑‑‑‑‑‑‑‑‑‑‑‑‑‑‑‑‑‑‑‑‑‑‑‑‑‑‑‑‑‑‑‑‑‑‑‑  >  NC_001604.1/943‑1252`  
`‑‑‑‑‑‑‑‑‑‑‑‑‑‑‑‑‑‑‑‑‑‑‑‑‑‑‑‑‑‑‑‑‑‑‑‑‑‑‑‑‑‑‑‑‑‑‑‑‑‑‑‑‑‑‑‑‑‑‑‑‑‑‑‑‑‑‑‑‑‑‑‑‑‑‑‑‑‑‑‑‑‑‑‑‑‑‑‑‑‑‑‑‑‑‑‑‑‑‑‑‑‑‑‑‑‑‑‑‑‑‑‑‑‑‑‑‑‑‑‑‑‑‑‑‑‑‑‑‑‑‑‑‑‑‑‑‑‑‑‑‑‑‑‑‑‑‑‑‑‑‑‑‑‑‑‑‑‑‑‑‑‑‑‑‑‑‑‑‑‑‑‑‑‑‑‑‑‑‑‑‑‑‑‑‑‑‑‑‑‑‑‑‑‑‑‑‑‑‑‑‑‑‑‑‑‑‑‑‑‑‑‑‑‑‑‑‑‑‑‑‑‑‑‑‑‑‑‑‑‑‑‑‑‑‑‑‑‑‑‑‑‑‑‑‑‑‑‑‑‑‑‑‑‑‑‑‑‑‑‑‑‑‑‑‑‑‑‑‑‑‑‑‑‑‑‑‑‑‑‑‑‑‑‑‑‑‑‑‑‑‑‑‑‑‑‑‑‑‑‑‑‑‑‑‑‑‑‑‑g‑‑‑‑‑‑‑‑‑a‑‑‑‑‑‑‑‑‑ggaagtcg‑‑‑‑‑‑‑C‑‑ACGACAGAAAGAAATTGACCGCGCTAAGGCCCGTAAAGAACGTCACGAGGGGCGCTTAGAGGCACGCAGATTCAAA‑CGTCGCAACCGCAAGGCACGTAAAGCACACAAAGCTAAGCGCGAAAGAATGCTTGCTGCGTGGCGATGGGCTGAACGTCAAGAACGGCGTAACCATGAGGTAGCTGTAGATGTACTAGGAAGAACCAATAACGCTATGCTCTGGGTCAACATGTTCTCTGGGGACTTTAAGGCGCTTGAGGAACGAATCGCGCTGCACTGGCGTAATGCTGACCGGATGGCT  >  NC_001604.1/2742‑3040`

  

Alignment Legend

Aligned base mismatch/match (shaded by quality score): `ATCG/ATCG` `< 3 ≤` `ATCG/ATCG` `< 10 ≤` `ATCG/ATCG` `< 22 ≤` `ATCG/ATCG` `< 34 ≤` `ATCG/ATCG` `< 38 ≤` `ATCG/ATCG`

Unaligned base: `atcg`    Masked matching base: `atcg`    Alignment gap: `‑`    Deleted base: `‑`

Reads not counted as support for junction

read\_name Not counted due to insufficient overlap past the breakpoint.

read\_name Not counted due to not crossing MOB target site duplication.
